# Supplementary figures and images for: Phylogenomic curation of Ovate Family Proteins (OFPs) in the U’s Triangle of Brassica L. indicates stress-induced growth modulation
Source: PLoS One. 2024 Jan 26;19(1):e0297473. doi: 10.1371/journal.pone.0297473 (PMC10817133; doi:10.1371/journal.pone.0297473)

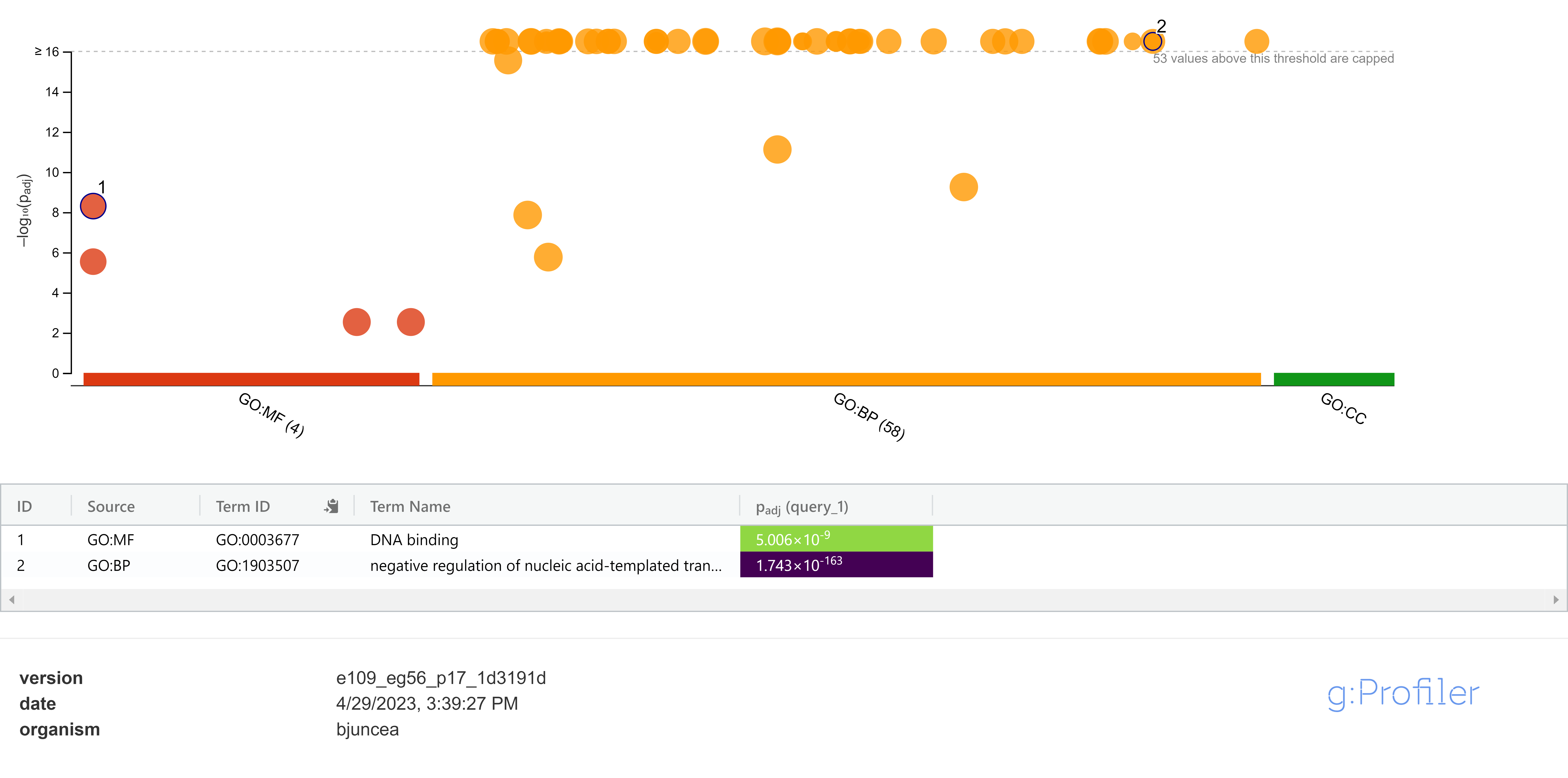

Supplement: S1 Dataset — (ZIP) [file pone.0297473.s009.zip › S1 Dataset/GO & KEGG/B. juncea/Bju_1.png]

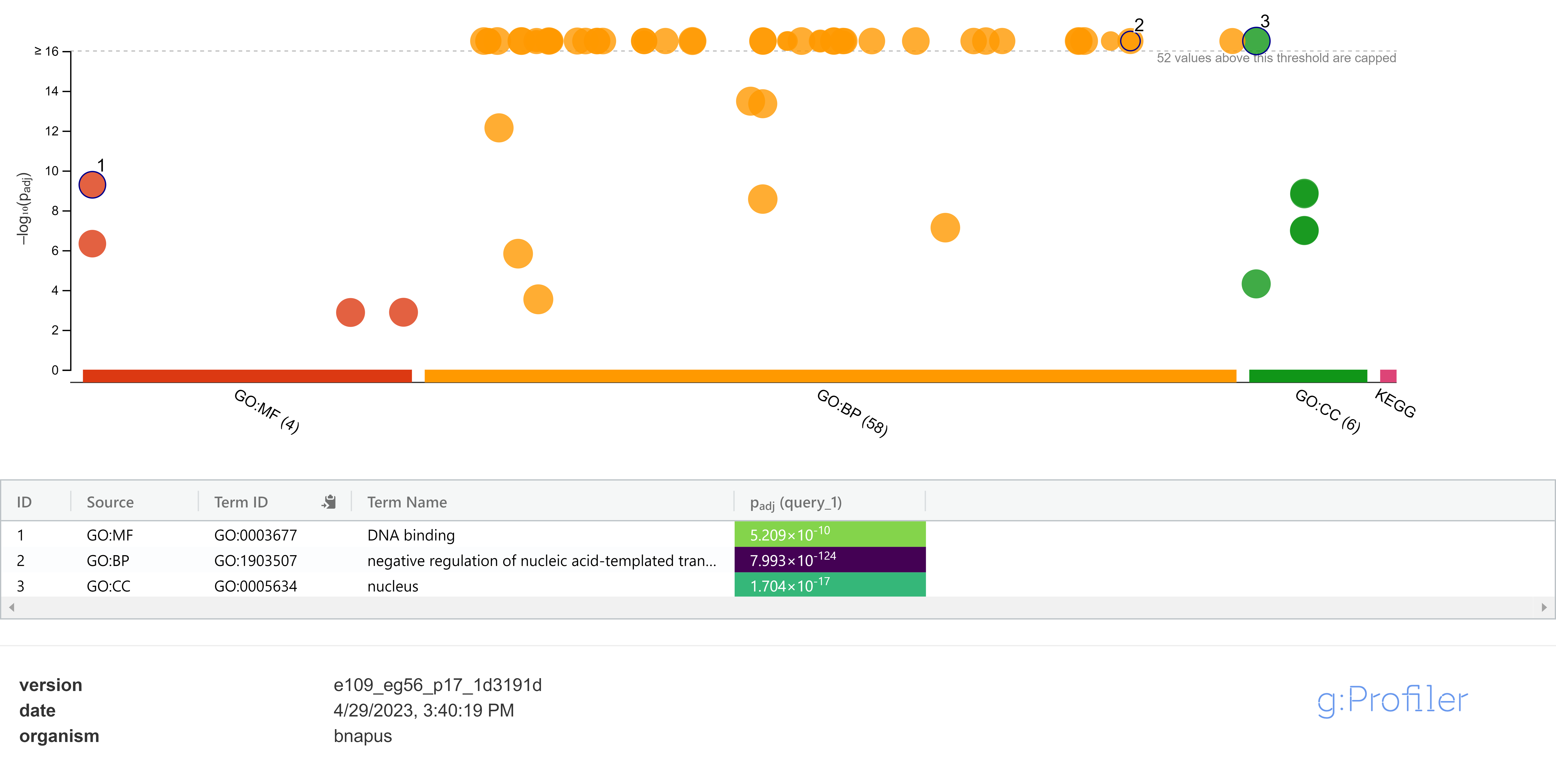

Supplement: S1 Dataset — (ZIP) [file pone.0297473.s009.zip › S1 Dataset/GO & KEGG/B. napus/Bna_1.png]

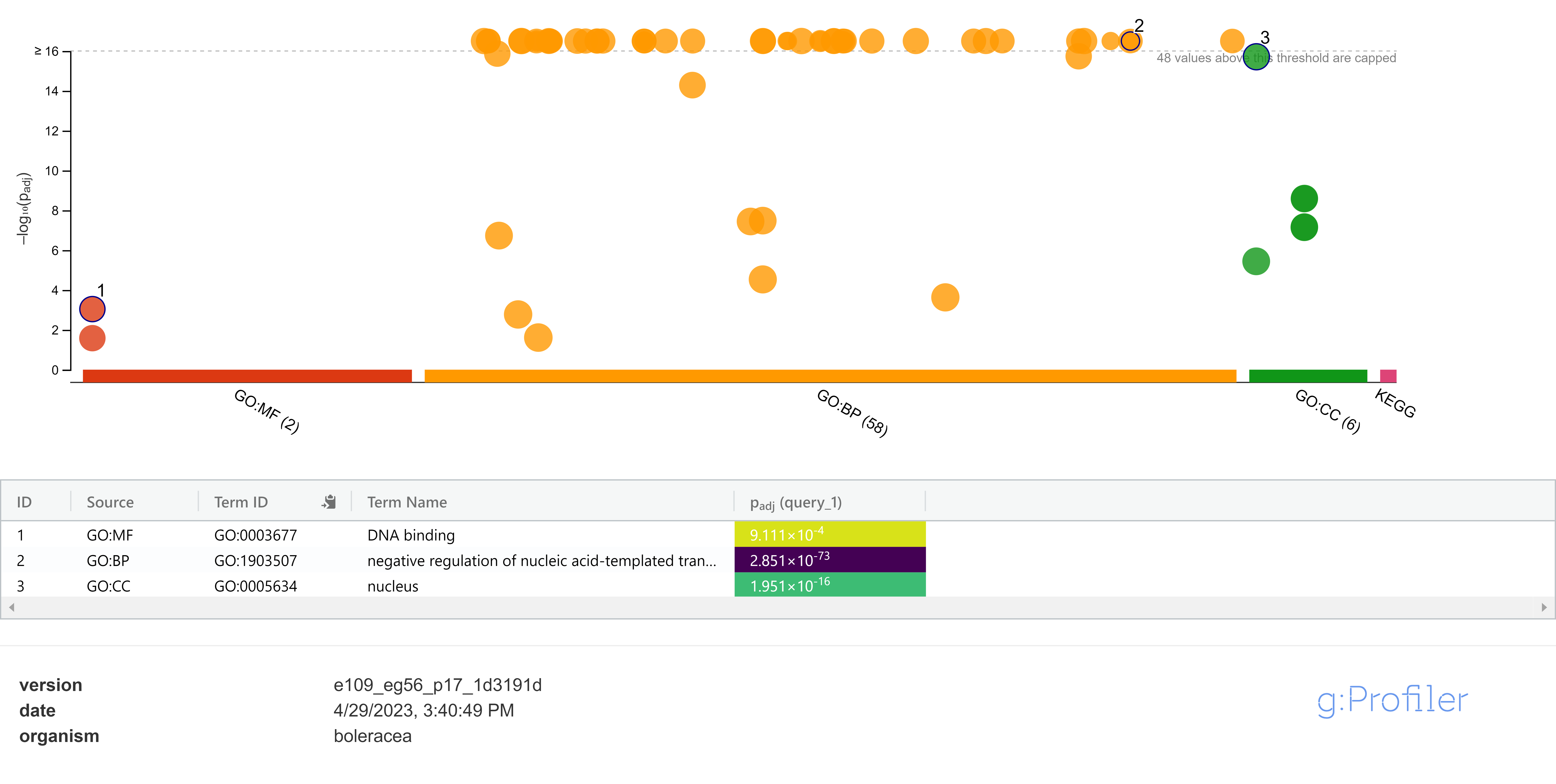

Supplement: S1 Dataset — (ZIP) [file pone.0297473.s009.zip › S1 Dataset/GO & KEGG/B. oleracea/Bol_1.png]

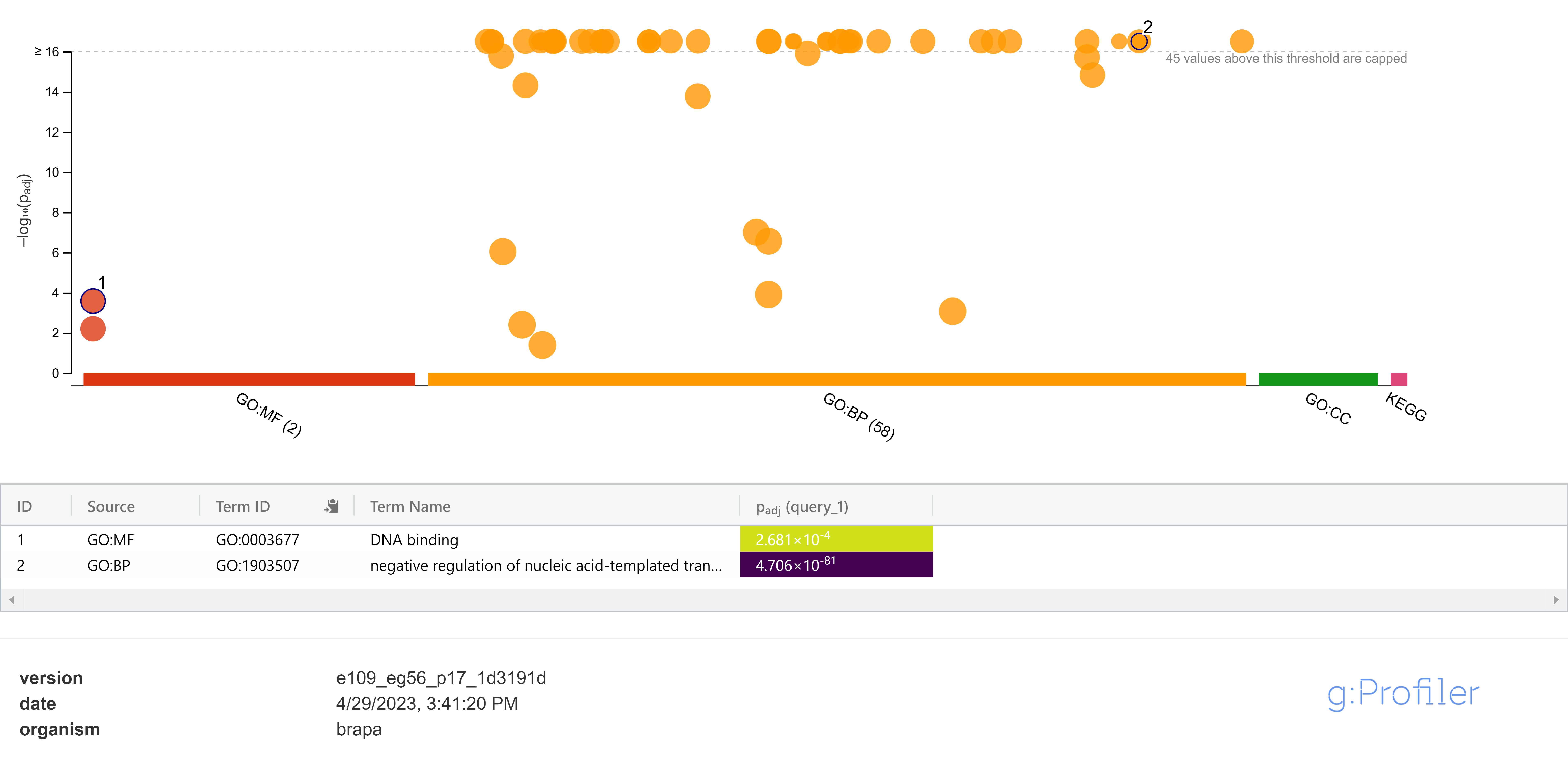

Supplement: S1 Dataset — (ZIP) [file pone.0297473.s009.zip › S1 Dataset/GO & KEGG/B. rapa/Bra_1.png]

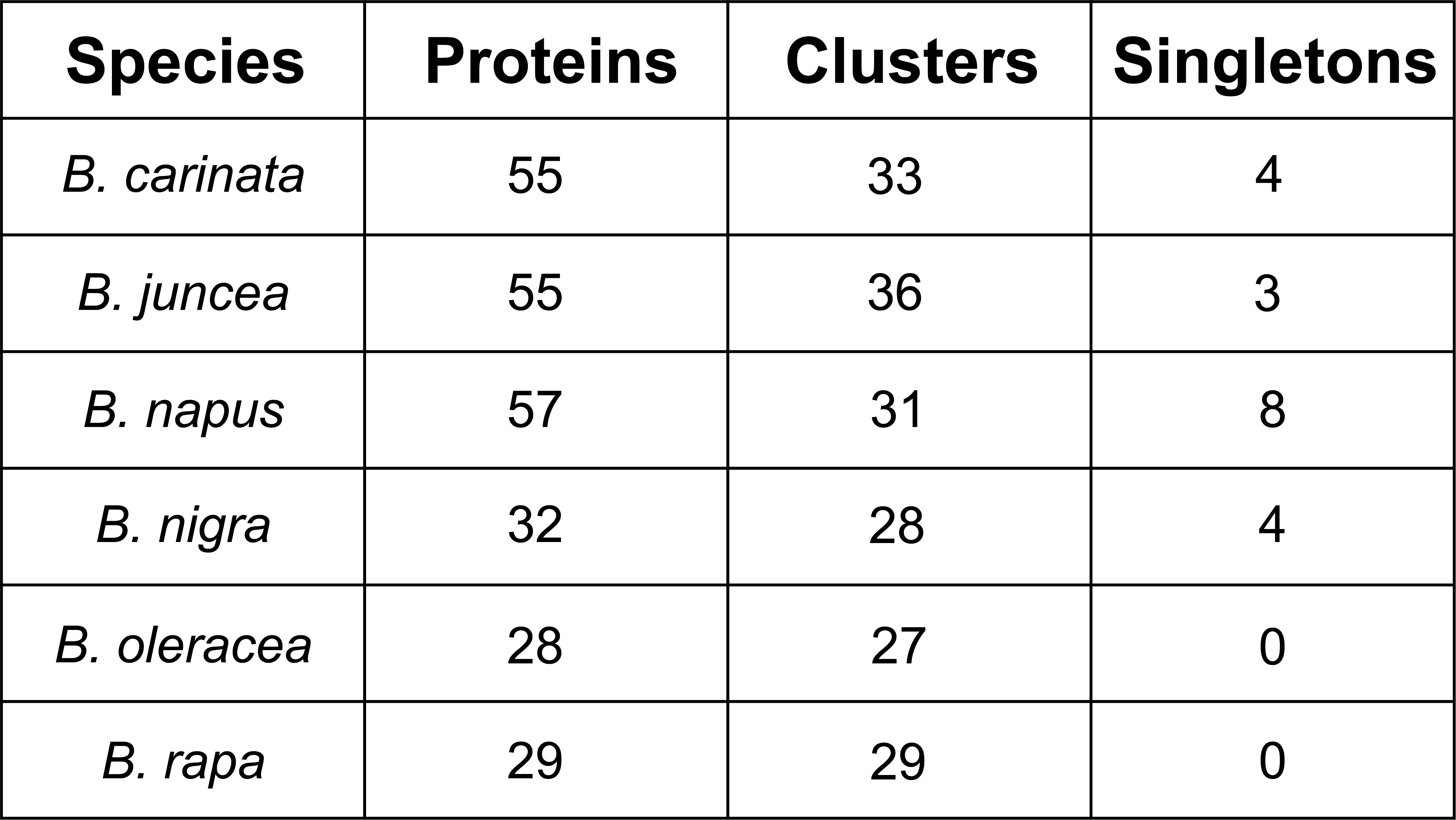

Supplement: S1 Dataset — (ZIP) [file pone.0297473.s009.zip › S1 Dataset/Orthology/OthoVenn_Info_U's Triangle.png]

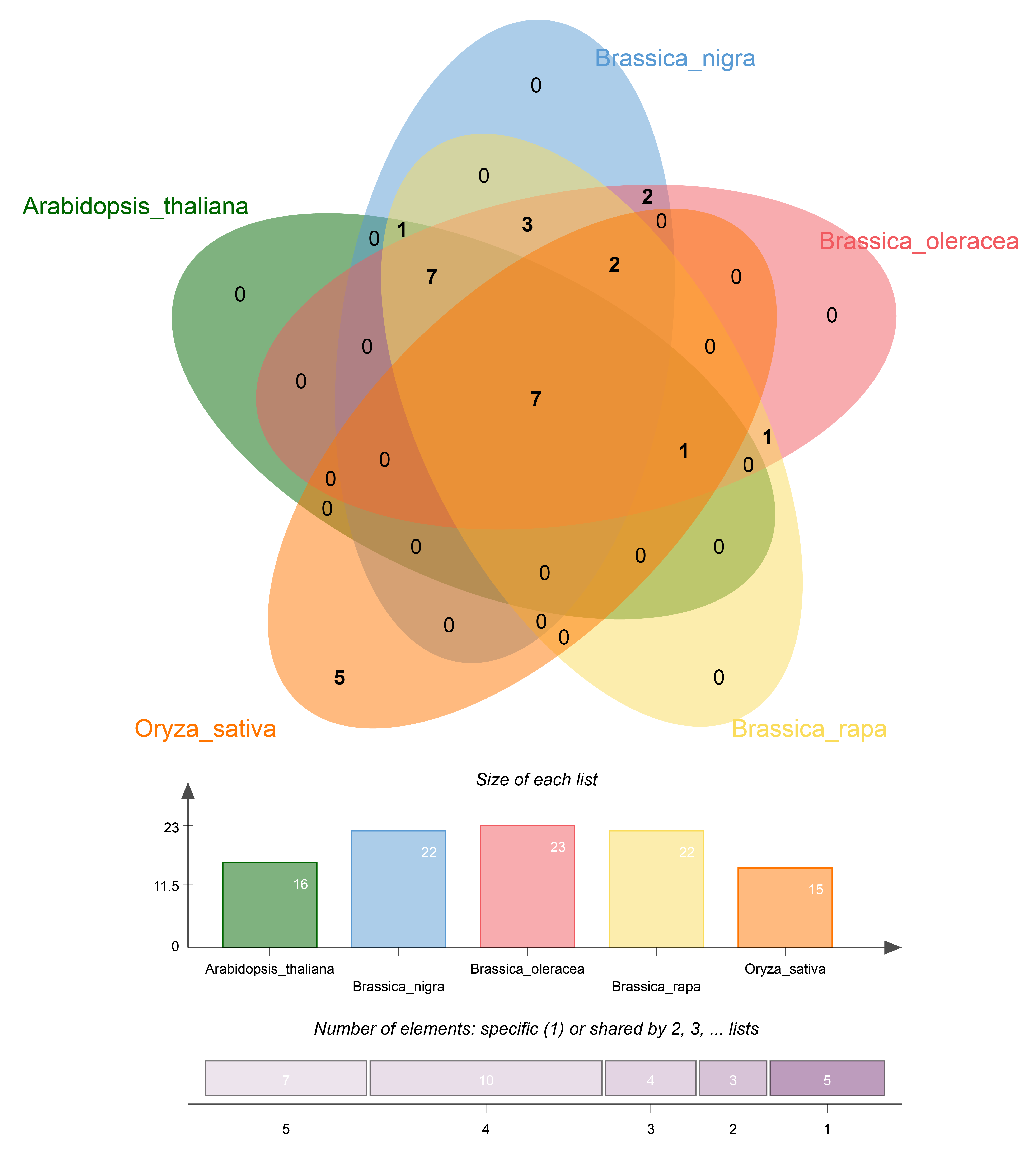

Supplement: S1 Dataset — (ZIP) [file pone.0297473.s009.zip › S1 Dataset/Orthology/Parents_At_Os.png]

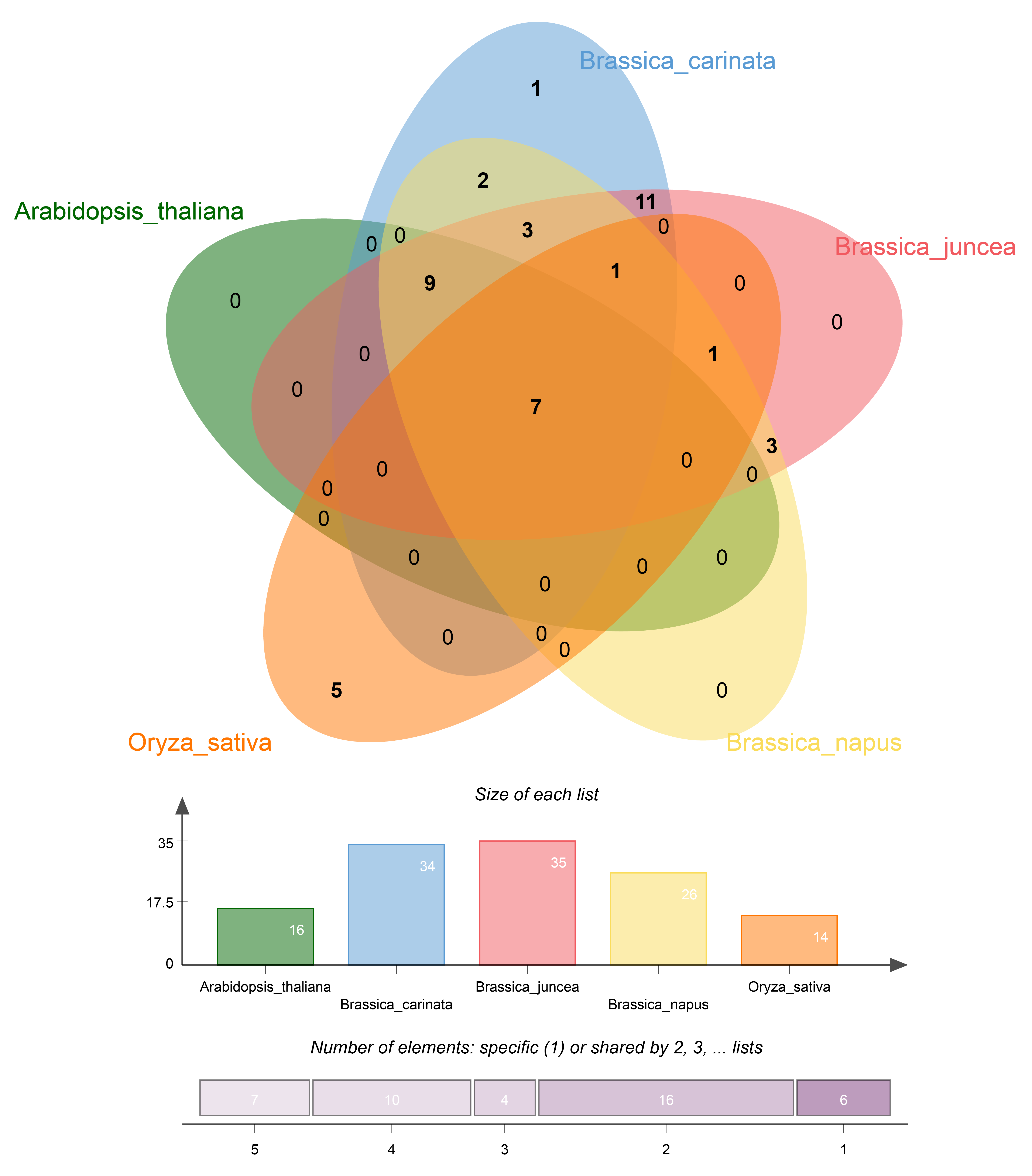

Supplement: S1 Dataset — (ZIP) [file pone.0297473.s009.zip › S1 Dataset/Orthology/Progeny_At_Os.png]

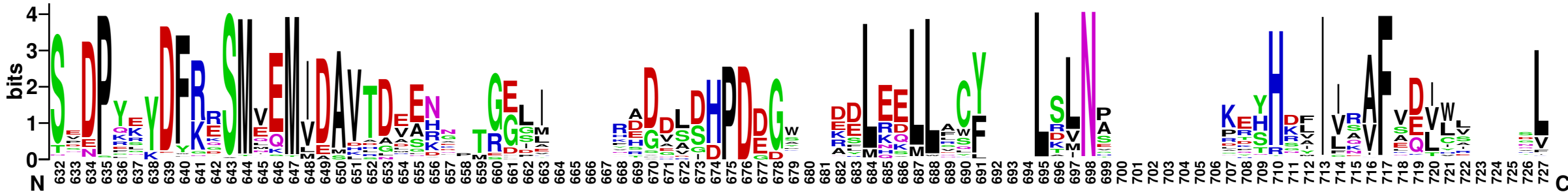

Supplement: S1 Dataset — (ZIP) [file pone.0297473.s009.zip › S1 Dataset/Sequence Logos/OVATE_Domain_Sequence_Logo.pdf]

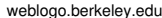

Supplement: S1 Dataset — (ZIP) [file pone.0297473.s009.zip › S1 Dataset/Sequence Logos/OVATE_Domain_Sequence_Logo_Frquency Plot.pdf]
