# Supplementary material for: Phylogenomic curation of Ovate Family Proteins (OFPs) in the U’s Triangle of Brassica L. indicates stress-induced growth modulation
Source: PLoS One. 2024 Jan 26;19(1):e0297473. doi: 10.1371/journal.pone.0297473 (PMC10817133; doi:10.1371/journal.pone.0297473)
Supplement: Attachment — Submitted filename: Response to Reviewers.pdf [file pone.0297473.s010.pdf]

Comment 5: Is the manuscript presented in an intelligible fashion and written in standard English?

Reviewer #2: No

- Author Response: Thanks for the recommendation. We've realized this mistake and this time we hired a native English copywriter to edit the manuscript from top to bottom. In the tracked changes version of the manuscript, you can see that all unnecessary information has been removed, the structure of the manuscript has been dynamically re-adjusted, and the sentences were corrected in accordance with the standard English style. We hope that this version of the manuscript will be right up there according to the publishing standards of PLoS ONE.

Comment 6: ………………………………

Reviewer #2: The study is valuable and important but the analysis is purely theoretical. This study needs to be validated but additional experiments.

- We've realized this shortcoming of the study as well. At the time of the previous submission, we did not have the resources to perform the additional validation experiments. But now, we have experimentally validated the findings through a series of qRT-PCRs under various conditions. We are confident that these validation experiments have improved the soundness of the manuscript as well as its practical reproducibility. Hopefully, this version is up to the standards of publication in PLoS ONE.
