# Supplementary material for: Phylogenomic curation of Ovate Family Proteins (OFPs) in the U’s Triangle of Brassica L. indicates stress-induced growth modulation
Source: PLoS One. 2024 Jan 26;19(1):e0297473. doi: 10.1371/journal.pone.0297473 (PMC10817133; doi:10.1371/journal.pone.0297473)
Supplement: Attachment — Submitted filename: Comments PLos One.pdf [file pone.0297473.s011.pdf]

Material and Method Section

In current study, there are many RNA samples and every RNA sample must have different concentration. Please elaborate how you made sure that the extracted RNA is of good quality. May be gel image of some of the extracted RNA, nanodrop image or concentration will showcase the good quality RNA. Secondly, if RNA every sample have different concentration, then why the authors took 1 µg of RNA to go for transcription? Did you quantify each sample ?

What the fragment size of the targeted genes used in qRT-PCR experiment? And what PCR conditions were used to run the experiment?

The authors claimed OFPs contains conserved domain. It is suggested that "Sequence Logos" may added to further check about the conserveness of the gene family.

Results:

Currently the images are not very clear. It is suggested that DPI of all images need to be upgraded. Each figure/image should have at least 300 DPI.

Error bars in BnaOFP13 and BnaOFP29 (Figure 8) need to be rechecked.
