# Supplementary material for: Phylogenomic curation of Ovate Family Proteins (OFPs) in the U’s Triangle of Brassica L. indicates stress-induced growth modulation
Source: PLoS One. 2024 Jan 26;19(1):e0297473. doi: 10.1371/journal.pone.0297473 (PMC10817133; doi:10.1371/journal.pone.0297473)
Supplement: Attachment — Submitted filename: Response to Reviewers.pdf [file pone.0297473.s012.pdf]

To Reviewer #1

Thanks for the positive feedback. We are truly grateful for the insightful revisions that you suggested. We have given each of your comment careful thought and have made the changes to the manuscript accordingly. Our humble response to your comments is given below.

1) Please put genes in italics name throughout the text.

> All the genes names have been edited in italic throughout the manuscript as requested.

2) Please use the Kobas database at http://bioinfo.org/kobas/annotate/ in the gene ontology section because it examines more details as compared to gProfiler.

> Thanks for this suggestion. We realized our shortcomings in the GO & KEGG analysis, so it has been revamped using the more robust KOBAS database as requested. Moreover, the output from the KOBAS-i module have been visualized using the SRplot utility to show the enrichment count of all the significant metabolic pathways. We think that you'll like the new KOBAS-mediated illustrations. Thanks again…

3) Please use Graphpad Parsim software to show the low value in the graph drawing section in Figure 8. Display the legend for each chart.

> Thanks for the suggestion. All the graphs that were previously developed using Excel have been redeveloped using the GraphPad Prism's latest version. All the low values have also been highlighted throughout using the numerical view function. All the missing legends have also been added. We hope you'll like the final results.

To Reviewer #2

Material and Method Section

In current study, there are many RNA samples and every RNA sample must have different concentration. Please elaborate how you made sure that the extracted RNA is of good quality. May be gel image of some of the extracted RNA, nanodrop image or concentration will showcase the good quality RNA. Secondly, if RNA every sample have different concentration, then why the authors took 1 µg of RNA to go for transcription? Did you quantify each sample ?

> Thanks for the comment. You're absolutely right. Each of the sample have different RNA concentrations and to make sure it's good quality RNA, we quantified it through a NanoDrop spectrophotometer. These values have now been mentioned in 'S8 Table' as desired. Moreover, three technical replicates further improved the quantification outcomes. On the other hand, each sample was quantified separately as mentioned above. In each template for the cDNA synthesis, a 1000ng concentration was kept constant through precise RNA concentration calculations (1000 / x- ngμL^{-1} of RNA). After that, 2 µL of cDNA was used in the main reaction mixture (2X SYBR Green Master qPCR Mix: 10 µL, F-Primer: 0.5 µL, R-Primer: 0.5 µL, ddH_2O: 07 µL, cDNA: 2 µL, Total : 20 µL). We forgot to mention it earlier and now the 'Material and Methods' section dealing with qRT-PCR has been modified accordingly. We're thankful for this valuable insight and hope that you'll like the modifications.

What the fragment size of the targeted genes used in qRT-PCR experiment? And what PCR conditions were used to run the experiment?

> Thanks for this insight. The fragment sizes of each of the genes have been added in 'S8 Table' in front of each of the developed primers. In terms of the subsequent qRT-PCR conditions, the standard manufacturer protocols are as following:

Reverse Transcription (RT):

- 60°C for 45 minutes.

PCR Amplification:

- Initial denaturation: 95°C for 30 seconds.
- Denaturation: 95°C for 15 seconds.
- Annealing: 60°C for 30 seconds.
- Extension: 72°C for 45 seconds.
- Final denaturation: 95°C for 30 seconds.
- Repeat for 40 cycles.

These conditions and all the other materials that have been used in PCR have been duly mentioned in the manuscript as desired.

The authors claimed OFPs contains conserved domain. It is suggested that "Sequence Logos" may added to further check about the conserveness of the gene family.

> Thanks for the suggestion. We realized that the main image only highlights the conserved domain position so we've added a high-quality set of 'Sequence Logos in the "S1 Dataset" as desired. This will give readers more context about the conserveness of the *OVATE* domain. We hope you'll like this addition. Thanks again…

Results:

Currently the images are not very clear. It is suggested that DPI of all images need to be upgraded. Each figure/image should have at least 300 DPI.

> Initially, all the vector images were rendered in 2400 dpi with sizes reaching > 100 Mb. In accordance with the journal submission guidelines, the maximum size is limited to < 10 Mb. Given your suggestion, we've reassessed and readjusted all the images for better visualization. It can also be due to the extreme PDF compression in the file that you're viewing from your point-of-view. We are confident these readjusted images will look better in the final production. Thanks again for the suggestion.

Error bars in BnaOFP13 and BnaOFP29 (Figure 8) need to be rechecked.

> Thanks for the suggestion. We realized this was a typo mistake on our side. We've corrected it and rechecked all other data for any other mistakes as well. We have also recreated the graphs in the Figure 8 using the latest version of GraphPad Prism. Even the low values have been numerically highlighted this time for a better experience to the reader. We hope you'll like the final results. Thanks again.

To Reviewer #3

Dear Reviewer,

We’re truly grateful for all your commentary and thankful that you liked our work. We’ve always strived toward robust researches that bring meaningful outcomes to the field. It feels euphoric when the significance of your hard work is appreciated by a person of the relevent field. Thanks again.

Regards,

Corresponding author
